# Long‐term retention on antiretroviral treatment after enrolment in prevention of vertical HIV transmission services: a prospective cohort study in Dar es Salaam, Tanzania

**DOI:** 10.1002/jia2.26186

**Published:** 2024-02-08

**Authors:** Roseline Faustine Urrio, Goodluck Willey Lyatuu, David Sando, Michael J. Mahande, Emmanuel Philipo, Helga Naburi, Peter Lyaruu, Amanda Kimonge, Kasasi Mayogu, Brenda Simba, Ayoub Muhamed Kibao, Michael Msangi, Zangin Zeebari, Gunnel Biberfeld, Anna Mia Ekström, Charles Kilewo, Anna E. Kågesten

**Affiliations:** ^1^ Management and Development for Health Dar es Salaam Tanzania; ^2^ Department of Obstetrics and Gynacology Muhimbili University of Health and Allied Sciences Dar es Salaam Tanzania; ^3^ Department of Global Public Health Karolinska Institutet Stockholm Sweden; ^4^ Department of Pediatric and Child Health Muhimbili University of Health and Allied Sciences Dar es Salaam Tanzania; ^5^ Office of the Regional Administrative Secretary Dar es Salaam Tanzania; ^6^ Ministry of Health Dodoma Tanzania; ^7^ Department of Infectious Disease/Venhälsan South General Hospital Stockholm Sweden

**Keywords:** vertical HIV transmission, antiretroviral treatment (ART), attrition, retention, pregnant/breastfeeding women, adolescent and young women

## Abstract

**Introduction:**

To prevent vertical HIV transmission and ensure healthy mothers and children, pregnant women with HIV must remain on antiretroviral treatment (ART) for life. However, motivation to remain on ART may decline beyond the standard 2‐year breastfeeding/postpartum period. We assessed attrition and retention in ART care among women with HIV up to 6 years since enrolment in vertical transmission prevention services in Dar es Salaam, Tanzania.

**Methods:**

A prospective cohort of 22,631 pregnant women with HIV were enrolled in vertical transmission prevention services between January 2015 and December 2017 in routine healthcare settings and followed‐up to July 2021. Kaplan−Meier was used to estimate time to ART attrition (died, stopped ART or was lost to follow‐up [no show ≥90 days since scheduled appointment]) and the proportion retained in care. Cox proportional hazard models were used to estimate adjusted hazard ratios (aHR) of ART attrition in relation to predictors.

**Results:**

Participants were followed‐up to 6 years for a median of 3 years (IQR: 0.1–4). The overall ART attrition rate was 13.8 per 100 person‐years (95% CI: 13.5–14.1), highest in the first year of enrolment at 27.1 (26.3–27.9), thereafter declined to 9.5 (8.9–10.1) in year 3 and 2.7 (2.1–3.5) in year 6. The proportion of women retained in care were 78%, 69%, 63%, 60%, 57% and 56% at 1, 2, 3, 4, 5 and 6 years, respectively. ART attrition was higher in young women aged <20 years (aHR 1.63, 95% CI: 1.38–1.92) as compared to 30‐39 year‐olds and women enrolled late in the third versus first trimester (aHR 1.29, 95% CI: 1.16–1.44). In contrast, attrition was lower in older women ≥40 years, women who initiated ART before versus during the index pregnancy and women attending higher‐level health facilities.

**Conclusions:**

ART attrition among women with HIV remains highest in the first year of enrolment in vertical transmission prevention services and declines markedly following a transition to chronic HIV care. Targeted interventions to improve ART continuity among women with HIV during and beyond prevention of vertical transmission are vital to ending paediatric HIV and keeping women and children alive and healthy.

## 1 INTRODUCTION

The rollout of universal lifelong antiretroviral treatment (ART) for all pregnant/breastfeeding women living with HIV (WLHIV), launched in 2012 as Option B+, made the global elimination of vertical HIV transmission an achievable goal [1, 2], and provided opportunities for better long‐term ART care and HIV prevention for WLHIV and their families. In 2021, 81% of 1.3 million pregnant WLHIV globally received ART to prevent vertical HIV transmission, a substantial increase from 45% in 2010 [3]. Lifelong ART for WLHIV during and after pregnancy reduces the vertical HIV transmission risk to <5% in breastfeeding and <2% in non‐breastfeeding populations, but also lowers the risks of HIV transmission to sexual partners and drug‐resistant viral mutations, and improves the health of WLHIV and their families [4, 5]. Nevertheless, suboptimal retention remains a major concern for WLHIV, ranging from 52% to 70% during the first 3 years postpartum [6, 7, 8].

Tanzania is among the 21 global plan countries prioritized for the prevention of vertical HIV transmission due to its persistently high (11%) transmission estimate [9]. In 2021, 1.7 million Tanzanians were living with HIV, of whom 59% were women of reproductive age (15–49 years) [10]. Tanzania has scaled up vertical transmission prevention services, with 97% of reproductive and child health (RCH) facilities offering ART to pregnant/breastfeeding women [9]. Tanzania adopted the universal lifelong ART strategy in 2013 and reached 80% ART coverage among pregnant/breastfeeding WLHIV in 2021 [10]. Nonetheless, retention on lifelong ART among pregnant/postpartum WLHIV remains a challenge, with loss to follow‐up (LTFU) as high as 52% [11]. HIV diagnosis during the index pregnancy, young age at ART initiation [12, 13], late third trimester ART initiation and receiving care through lower‐level rather than higher‐level health facilities affect ART retention [14]. While the transition from vertical transmission prevention services to chronic HIV care following the standard follow‐up of 2‐year postpartum may increase the risk of ART attrition [15], less is known about long‐term retention in ART care beyond this period of time in Eastern Africa including Tanzania.

We aimed to assess long‐term attrition and retention in ART care among a large cohort of WLHIV up to 6 years after enrolment in vertical transmission prevention services within routine healthcare settings in Dar es Salaam, Tanzania. We also investigated socio‐demographic and clinical predictors of ART attrition over time.

## 2 METHODS

### 2.1 Study design, setting and population

We conducted a prospective cohort study using data from routine healthcare records of WLHIV enrolled in vertical transmission prevention services and receiving lifelong ART between January 2015 and December 2017, and followed‐up until July 2021. This study was conducted in Dar es Salaam, with 5.3 million residents and an HIV prevalence of 6.3% among women of reproductive age, three times that of men at 2.0% [9]. Dar es Salaam (and Tanzania) has a hierarchical health facility structure composed of lower‐level dispensaries that provide outpatient primary care, health centres that provide outpatient and inpatient care, and hospitals that provide specialized outpatient and inpatient care [16]. In Dar es Salaam, antenatal care (ANC) is provided in 271 health facilities which enrol over 160,000 pregnant women annually. Most pregnant women in Dar es Salaam (99%) attend ANC at least once [9], and receive a comprehensive care package, including testing for HIV, syphilis, tuberculosis, and malaria, family planning, safer infant feeding and safer delivery counselling.

In Tanzania, all pregnant women diagnosed with HIV are enrolled in vertical transmission prevention services, including ART initiation or continuation for those already on ART. Women are issued unique HIV care and treatment clinic (CTC) identification numbers (ID) and followed‐up monthly at RCH clinics for integrated ANC/ART and Early Infant HIV Diagnosis (EID) services. At 2 years postpartum, upon receipt of final infant HIV test results or earlier for infants diagnosed with HIV, women are discharged from integrated ANC/ART to general HIV clinics to continue chronic HIV care. Upon transfer, women are uniquely identified by their CTC‐ID. To address ART adherence and retention, ANC/ART clinics have deployed peer mothers to provide ART psychosocial support and remind women of their clinic appointments and EID test dates. During the current study period, peer mother engagement was paused between April and October 2020 due to the COVID‐19 pandemic.

Study participants were identified across 226 public and private health facilities (with an accessible CTC2 database), which provide >90% of vertical transmission prevention services in Dar es Salaam. WLHIV, both those diagnosed with HIV before pregnancy and those newly diagnosed during the index pregnancy, enrolled on ART and vertical prevention services between January 2015 and December 2017 were included. The index pregnancy is the first pregnancy recorded during the enrolment period. Those who did not start ART were excluded.

### 2.2 Procedures

Participants were followed‐up from their first routine ANC clinic visits for the index pregnancy through transfer to general chronic HIV care clinics for the 2‐year postpartum period and again at ANC for any subsequent pregnancies during the follow‐up period. Socio‐demographic, clinical and laboratory monitoring information were collected using routine national patient charts and entered into the electronic CTC2 database that stores and manages Tanzania's ART and vertical HIV transmission data.

### 2.3 Measures

The primary study outcome was time to ART attrition (death, stopping ART or LTFU) [16]. Attrition due to death was recorded in the CTC2 database upon notification by the family or healthcare providers. Women who stopped ART were defined as those who, at last contact with the clinic, reported having stopped ART and not re‐started ART over 90 days. Women who were not recorded in the CTC2 database as having died, transferred out or stopped ART and who did not show up at the clinic for over 90 days after their previous appointment were classified as LTFU [17]. For women without a documented next appointment, the LTFU definition was defined as not showing up at the clinic for over 180 days following the previous visit, based on the maximum allowable appointment spacing in Tanzania's ART guidelines. Time to ART attrition was measured from the date of enrolment in vertical transmission prevention services to the last clinic visit. The cumulative proportion of women retained on ART after accounting for death, stopping ART, LTFU and transfers was also estimated as well as the proportion of women with repeated pregnancies, defined as a pregnancy occurring after the index pregnancy, during the follow‐up period.

The following socio‐demographic and clinical covariates were based on information extracted from the CTC2 database: age (<20, 20–29, 30–39, 40+ years), marital status (single, married/cohabiting, divorced/separated/widow), gestation age (first, second or third trimester), year of HIV (< 2015–2017), entry point at HIV diagnosis (outpatient/inpatient departments, RCH, voluntary counselling testing, others), advanced HIV disease status (yes vs. no based on WHO Clinical Stage III or IV or CD4 count <200 cells per microlitre [μl]), ART regimen core (first‐line [non‐nucleoside reverse transcriptase inhibitor] vs. second‐line [protease inhibitor]), ART status at enrolment in vertical transmission prevention services, health facility level (dispensary, health centre, hospital) and facility ownership (private/public).

### 2.4 Statistical analysis

Categorical variables were summarized by proportions, and continuous variables were categorized based on clinical relevance. The proportion of missing data ranged from 5.6% (age) to 25.7% (gestational age), likely due to random incomplete documentation. Multiple imputation for missing data was performed, assuming random missingness, using chained equations followed by repeated multivariable regression model to account for possible systematic differences between observed and missing data in complete case analysis.

Pearson's Chi‐square test tested bivariate associations between categorical covariates. Continuous variables were summarized by mean and standard deviation (SD) or median and interquartile range (IQR). Kaplan−Meier survival analysis estimated the time from enrolment in vertical transmission prevention services, to attrition (failure event). Women with no follow‐up visit, that is their only visit was at enrolment, were assigned a follow‐up time of 0.1 and included in the survival analysis. Annual retention was also estimated by Kaplan−Meier analysis. Study participants confirmed to have transferred to other facilities were censored on the transfer date and assumed to be on ART. All participants were censored at their last documented visit date.

Cox proportional hazards regression models accounting for clustering within health facilities were performed to estimate the adjusted hazard ratio (aHR) and corresponding 95% confidence intervals (CI) of ART attrition relative to socio‐demographic and clinical characteristics as potential predictors. Covariates were tested in crude and multivariable models. Variables with Wald test *p*‐value of ≤ 0.2 were initially included in the multivariable models and thereafter stepwise selected through backwards elimination for final adjusted models. Marital status was maintained in the model given the evidence that it predicts ART attrition [18]. Finally, evaluation of the assumption of proportionality of hazards on the basis of Schoenfeld residuals was not significant overall but for ART status at enrolment (*p* = 0.003). Therefore, sensitivity analyses via Cox regression was run for each stratum of ART status at enrolment and found to be consistent with the unstratified model. Here, the results of the unstratified model are presented based on the assumption of proportionality hazards. All analyses were performed using Stata Software version 14 (StataCorp. 2015. *Statistical Software: Release 14*. College Station, TX: StataCorp LP).

### 2.5 Ethical considerations

This study was approved by the Ethical Review Board of Muhimbili University of Health and Allied Sciences, “Ref. No. DA.282/298/01.C/020” in Tanzania and did not require approval by the National Ethics Board. Informed consent was waived since the study used anonymized data from electronic routine healthcare records.

## 3 RESULTS

### 3.1 Baseline characteristics

A total of 22,671 WLHIV from 226 Dar es Salaam health facilities who registered for vertical transmission prevention services between January 2015 and December 2017 were identified. Forty women with missing ART start dates were excluded leaving 22,631 women (Table 1).

**Table 1:** Baseline and clinical characteristics at enrolment among pregnant women enrolled in universal lifelong ART from January 2015 to December 2017 in routine healthcare setting of Dar es Salaam, Tanzania (*N* = 22,631)

Patient characteristics	Total (*N* = 22,631)	Started ART during index pregnancy (*N* = 14,238)	Started ART before index pregnancy (*N* = 8393)	*p*‐Value
	*n* (column %)	*n* (row %)	*n* (row %)	
**Age (years)**				
<20	867 (3.8)	701 (80.8)	166 (19.2)	
20–29	10,128 (44.8)	7544 (74.5)	2584 (25.5)	
30–39	10,375 (45.8)	5503 (53.0)	4872 (47.0)	
40+	1261 (5.6)	490 (38.9)	771 (61.1)	0.001
**Marital status**				
Single	5347 (23.6)	2871 (53.7)	2476 (46.3)	
Married/cohabiting	11,863 (52.4)	7748 (65.3)	4115 (34.7)	
Divorced/separated/widow	814 (3.6)	243 (29.8)	571 (70.2)	
Missing	4607 (20.4)	3376 (73.3)	1231 (26.7)	0.001
**Gestational age, trimester**				
First	2964 (13.1)	1591 (53.7)	1373 (46.3)	
Second	10,786 (47.7)	7586 (70.3)	3200 (29.7)	
Third	3067 (13.5)	2022 (65.9)	1045 (34.1)	
Missing	5814 (25.7)	3039 (52.3)	2775 (47.7)	0.001
**Year of HIV diagnosis**				
<2015	7298 (32.3)	699 (9.6)	6599 (90.4)	
2015	5330 (23.5)	4522 (84.8)	808 (15.2)	
2016	4966 (21.9)	4308 (86.7)	658 (13.3)	
2017	5037 (22.3)	4709 (93.5)	328 (6.5)	0.001
**Advanced HIV disease**				
No	16,810 (74.3)	13,029 (77.5)	3781 (22.5)	
Yes	5811 (25.7)	1201 (20.7)	4610 (79.3)	
Missing	10 (0.04)	8 (80)	2 (20)	0.001
**ART regimen core**				
NNRTI (first line)	22,412 (99.0)	14,225 (63.5)	8187 (36.5)	
Protease inhibitor (second line)	219 (1.0)	13 (5.9)	206 (94.1)	0.001
**Entry point at HIV diagnosis**				
Out‐patient and in‐patient departments	2119 (9.4)	855 (40.4)	1264 (59.6)	
RCH	12,354 (54.6)	10,475 (84.8)	1879 (15.2)	
Voluntary counselling and testing	4581 (20.2)	1173 (25.6)	3408 (74.4)	
Others	858 (3.8)	226 (26.3)	632 (73.7)	
Missing	2719 (12.0)	1509 (55.5)	1210 (44.5)	0.001
**Repeated pregnancy**				
No	18,879 (83.4)	12,266 (65.0)	6613 (35.0)	
Yes	3752 (16.6)	1972 (52.6)	1780 (44.4)	0.001
**Health facility type/level**				
Dispensary	12,725 (56.2)	8532 (67.1)	4193 (32.9)	
Health centre	6211 (27.4)	3957 (63.7)	2254 (36.3)	
Hospital	3695 (16.3)	1749 (47.3)	1946 (52.7)	0.001
**Health facility ownership**				
Private	4898 (21.6)	3093 (63.2)	1805 (36.8)	
Public	17,733 (78.4)	11,145 (62.8)	6588 (37.2)	0.001

**Abbreviations**: ART, antiretroviral treatment; HIV, human immunodeficiency virus; NNRT, non‐nucleoside reverse transcriptase inhibitor; RCH, reproductive and child health.

Study participants were followed‐up until July 2021, with a median follow‐up time from enrolment of 3 years (IQR: 0.1–4) and a maximum of 6 years. The mean age at enrolment was 29.8 (SD 6.2) years. Most (82.4%) were enrolled in vertical transmission prevention services late (second or third trimester of pregnancy). About one‐third (37.1%) had been diagnosed with HIV and initiated lifelong ART prior to enrolment.

### 3.2 ART attrition and retention

**Figure 1:**
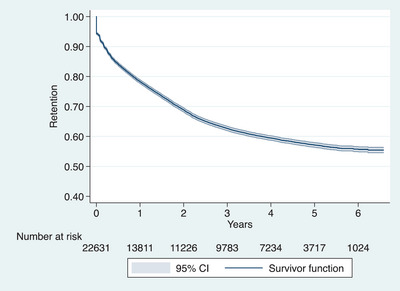
Kaplan−Meier estimates of retention in ART care for 22,631 women who were enrolled in vertical HIV transmission prevention services during pregnancy from January 2015 to December 2017 and followed‐up to July 2021 in Dar es Salaam, Tanzania (*N* = 22,631).

A total of 7714 attrition events were observed (176 stopped ART, 363 deaths and 7175 LTFU) over 55,732 person‐years of follow‐up, equivalent to an attrition rate of 13.8 per 100 person‐years (95% CI: 13.5–14.1). Table 2 and Figure 1 describe the Kaplan−Meier estimates of the annual attrition rate and proportion of women retained during each year of follow‐up. Over half of the attrition events (4374, 57%) occurred during the first year representing the highest attrition rate: 27.1 per person‐years (95% CI, 26.3–27.9), declining by over half to 12.8 (12.2–13.4) in the second year, and thereafter continued to decline by 23%–44% per year to 2.7 (2.1–3.5) in the sixth year. The majority of the 4374 first‐year attritions were driven by 2068 (47%) women who made only one visit and never returned.

**Table 2:** Annual attrition rate per 100 person‐years of follow‐up and annual retention rates among women on ART who were enrolled in vertical HIV transmission prevention services care during pregnancy in Dar es Salaam, Tanzania (*N* = 22,631)

Year	# retained	# attritions	Attrition rate/100 py (95% CI)	% Retained (95% CI)
1 year	13,819	4374	27.1 (26.3−27.9)	78.3 (77.7−78.8)
2 years	11,233	1598	12.8 (12.2−13.4)	68.9 (68.2−69.5)
3 years	9787	991	9.5 (8.9−10.1)	62.7 (61.9−63.4)
4 years	7234	467	5.3 (4.8−5.8)	59.5 (58.7−60.2)
5 years	3723	218	4.1 (3.5−4.6)	57.1 (56.4−57.9)
6 years	1036	63	2.7 (2.1−3.5)	55.7 (54.9−56.6)

Abbreviations: ART, antiretroviral treatment; CI, confidence interval; py, person‐years.

Similarly, the annual proportion of women retained dropped sharply to 78.3% (95% CI: 77.7%–78.8%) in the first year of follow‐up and thereafter declined by 1.4%–9.4% percentage points per year to 55.7% (95% CI: 54.9–56.6) in year 6 (Figure 1).

**Figure 2:**
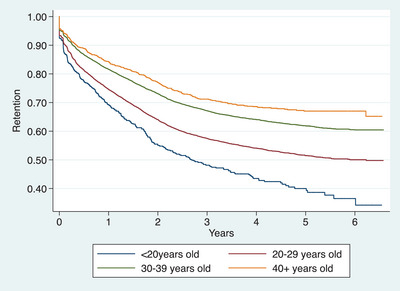
Kaplan−Meier estimates of retention in ART care for women enrolled in vertical HIV transmission prevention services during pregnancy from January 2015 to December 2017 and followed‐up to July 2021 in Dar es Salaam, Tanzania, stratified by age at antenatal care enrolment (*N* = 22,631).

**Figure 3:**
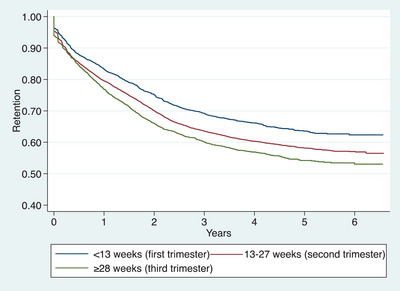
Kaplan−Meier estimates of retention in ART care for women enrolled in vertical HIV transmission prevention services during pregnancy from January 2015 to December 2017 and followed‐up to July 2021 in Dar es Salaam, Tanzania, stratified by gestational age at antenatal care enrolment (*N* = 22,631).

**Figure 4:**
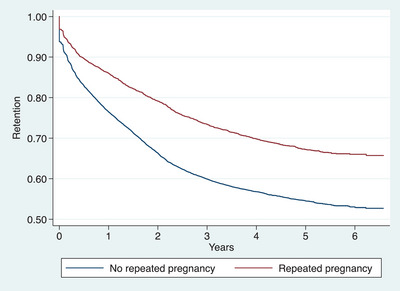
Kaplan−Meier estimates of retention in ART care for women enrolled in vertical HIV transmission prevention services during pregnancy from January 2015 to December 2017 and followed‐up to July 2021 in Dar es Salaam, Tanzania, stratified by repeated pregnancy during follow‐up period (*N* = 22,631).

At the end of follow‐up, cumulative ART retention decreased with lower maternal age, whereby only 36.5% of adolescents aged <20 years were retained in ART care, compared to 50%, 60% and 67% of women aged 20–29, 30–39 and 40+ years, respectively (Figure 2). ART retention also decreased with increasing gestation age at enrolment, only 53% of women enrolled for vertical transmission prevention services in their third trimester were retained, compared to 57% in their second and 62% in their first trimester (Figure 3). Furthermore, repeated pregnancy was associated with ART retention, with 53% of women with no repeated pregnancy retained compared to 66% with repeated pregnancy (Figure 4).

### 3.3 Predictors of ART attrition

Age, gestational age, ART status at enrolment, year of HIV diagnosis, advanced HIV disease, repeated pregnancy and health facility level at enrolment were all significant predictors of ART attrition (Table 3). Adolescents aged <20 years had a 63% higher attrition rate (aHR 1.63, 95% CI: 1.38–1.92) than 30‐ to 39‐year‐old women; and attrition was 12%–29% higher for women enrolled in their second trimester (aHR 1.12, 95% CI: 1.02–1.22) and third trimester (aHR 1.29, 95% CI: 1.16–1.44), respectively, compared to the first trimester. Women who started ART before versus during the index pregnancy had a 31% lower attrition rate (aHR 0.69, 95% C1: 0.54–0.59). Similarly, women enrolled in ART care with advanced HIV disease had a 12% lower attrition rate (aHR 0.88, 95% CI: 0.81–0.93) than those without. Women who had a repeated pregnancy during the follow‐up period had a 35% lower attrition rate (aHR 0.65, 95% CI: 0.60–0.71) than those who did not. Finally, attrition was 16% lower among women receiving ART care in higher‐level health centres (aHR 0.84, 95% C1: 0.77–0.90) than in lower‐level dispensaries. Marital status, ART regimen, point of entry for HIV diagnosis or health facility ownership were not significantly associated with attrition.

**Table 3:** Predictors of attrition among women on ART who were enrolled in vertical HIV transmission prevention services during pregnancy from January 2015 to December 2017 and followed‐up to July 2021 in Dar es Salaam, Tanzania (*N* = 22,631)

Patient characteristics	Crude HR (95% CI) *N* = 22,631	*p*‐Value	Adjusted HR (95% CI) *N* = 11,991	*p*‐Value	Multiple imputation adjusted HR (95% CI)	*p*‐Value
**Age (years)**						
<20	1.93 (1.71−2.18)	0.001	1.63 (1.38−1.92)	0.001	1.67 (1.48−1.90)	0.001
20–29	1.40 (1.33−1.47)	0.001	1.26 (1.17−1.35)	0.001	1.24 (1.18−1.31)	0.001
30–39	1[referent]		1[referent]		1[referent]	
40+	0.83 (0.74−0.94)	0.002	0.83 (0.70−0.98)	0.027	0.89 (0.79−1.00)	0.062
**Marital status**						
Single	1.03 (0.98−1.09)	0.262	1.03 (0.96−1.11)	0.412	1.05 (0.99−1.12)	0.069
Married/cohabiting	1[referent]		1[referent]		1[referent]	
Divorced/separated/widowed	0.93 (0.83−1.05)	0.260	1.21 (1.03−1.41)	0.018	1.17 (1.03−1.32)	0.012
**Gestational age, trimester**						
First	1[referent]		1[referent]		1[referent]	
Second	1.21 (1.13−1.30)	0.001	1.12 (1.03−1.22)	0.011	1.05(0.99−1.12)	0.140
Third	1.37 (1.25−1.49)	0.001	1.29 (1.16−1.44)	0.001	1.17 (1.03−1.32)	0.001
**Year of HIV diagnosis**						
<2015	1[referent]		1[referent]		1[referent]	
2015	1.76 (1.65−1.87)	0.001	1.24 (1.10−1.39)	0.001	1.21 (1.11−1.33)	0.001
2016	1.72 (1.61−1.83)	0.001	1.16 (1.03−1.32)	0.012	1.24 (1.13−1.36)	0.001
2017	2.05 (1.92−2.18)	0.001	1.28 (1.13−1.46)	0.001	1.37 (1.24−1.51)	0.001
**Advanced HIV disease**						
Yes	0.65 (0.61−0.68)	0.001	0.88 (0.81−0.93)	0.002	0.93 (0.83−0.99)	0.021
No	1[referent]		1[referent]		1[referent]	
**ART status at enrolment**						
Already on ART	0.56 (0.54−0.59)	0.001	0.69 (0.62−0.76)	0.001	0.71(0.65−0.77)	0.001
Started ART at first ANC visit	1[referent]		1[referent]			
**ART regimen core**						
NNRTI (first line)	1[referent]					
Protease inhibitor (second line)	0.68 (0.52−0.88)	0.004				
**Entry point at HIV diagnosis**						
Out‐patient and in‐patient departments	0.76 (0.70−0.83)	0.001				
RCH	1[referent]					
Voluntary counselling testing	0.69 (0.65−0.74)	0.001				
Others	0.70 (0.62−0.79)	0.001				
**Repeated pregnancy**						
No	1[referent]		1[referent]		1[referent]	
Yes	0.63 (0.60−0.67)	0.001	0.65 (0.60−0.71)	0.001	0.63 (0.60−0.67)	
**Health facility type/level**						
Dispensary	1[referent]		1[referent]		1[referent]	
Health centre	0.79 (0.76−0.84)	0.001	0.84 (0.77−0.90)	0.001	0.81(0.76−0.86)	0.001
Hospital	0.88 (0.82−0.93)	0.001	0.98 (0.90−1.07)	0.700	1.01(0.95−1.08)	0.687
**Health facility ownership**						
Private	1[referent]					
Public	0.95 (0.90−1.00)	0.051				

**Abbreviations**: ART, antiretroviral treatment; HIV, human immunodeficiency virus; NNRTI, non‐nucleoside reverse transcriptase inhibitor; RCH, reproductive and child health.

## 4 DISCUSSION

To the best of our knowledge, this is the first study to assess ART attrition and retention up to 6 years in a large cohort of WLHIV who were enrolled in universal lifelong ART for the prevention of vertical HIV transmission in Eastern Africa. Just over half (56%) of the women enrolled on ART were retained on treatment following the transition from vertical transmission prevention services to chronic HIV care. This low retention rate challenges UNAIDS’ goals to end the HIV epidemic by 2030 by reducing HIV mortality, morbidity and transmission.

Our study confirms and extends previous evidence on women at risk of discontinuing lifelong ART after vertical transmission prevention services. The first year of enrolment in vertical transmission prevention services had the highest attrition rate, comparable to other studies [8, 19], indicating the need for extra support. Potential reasons for high initial attrition include difficulties coping with one's HIV diagnosis, fear of stigma and discrimination, and lack of support for ART‐related side‐effects [20, 21]. Distance to the clinic combined with transportation costs are also barriers to retention [22]. The high proportion (9.1%) of women not returning after their first visit is also reported previously from Tanzania [11] and Mozambique [19], where one‐quarter of pregnant women dropped out after enrolment. This suggests that more active counselling is needed at the first ART visit for WLHIV on the importance of remaining on lifelong ART. Early engagement with treatment support groups (community and facility), as well as financial and emotional support from family members, can improve care engagement among WLHIV in their first year of enrolment [23].

The high attrition rates in the second and third year from enrolment also confirm previous findings [24], and may be due to a loss of motivation to continue ART once an infant has been declared HIV negative, as reported by the Tanzania Mitra Plus Study [25], and asymptomatic women not viewing themselves as needing lifelong treatment [26]. Transitioning to a general HIV clinic with new staff may also contribute to attrition [27]. Long‐term ART engagement requires repeated individually tailored counselling sessions [21, 23], active monitoring and management of ART‐related side‐effects, consistent family and peer support [28], supporting the transition into chronic HIV care, and providing multi‐month scripting for stable women better aligned with child immunization appointments [22].

In line with studies with shorter follow‐up, adolescents [13, 29], women who started ART late [15], or at lower‐level facilities, and those without a subsequent pregnancy had higher attrition [14, 30]. Our findings provide important evidence that these factors remain associated with long‐term ART attrition.

The high adolescent ART attrition may be explained by social, cultural, developmental and economic factors. Young women often depend on their parents or male partners for financial support and decisions in vertical transmission prevention services. The stigma surrounding premarital sex and teenage pregnancy, lack of HIV/ART knowledge [31] and lack of youth‐friendly health services are other known barriers to engaging adolescents in ART care [32, 33]. Adolescents are also more likely than older women to start ART during or late in pregnancy, as was the case in our study, compounding the risk of poor retention [6]. Our findings suggest more person‐centred and adolescent‐friendly HIV services to promote routine testing for early ART initiation and optimize existing vertical transmission prevention services to address the needs of adolescent girls and young women and keep them in care.

Consistent with other studies, women starting ART before pregnancy had higher retention compared to those starting during pregnancy [6, 34]. Most likely, these women had more time to accept their HIV diagnosis and prepare for lifelong treatment [35]. Our finding that women starting ART in their third trimester had higher attrition confirms the vulnerability of late ANC presenters, which may result in inadequate adherence counselling before delivery or common social determinants predicting both delay in healthcare seeking and low retention [36, 37].

Similar to other East African and Haitian studies, women enrolled with advanced HIV disease had lower attrition [38, 39]. These women may be more concerned about their HIV acquisition, with stronger incentives to become healthier and live longer, resulting in better care‐seeking behaviours [40]. Healthy, symptom‐free women may consider themselves too physically fit to seek care until they feel sick [40].

Contrary to other studies, women accessing ART in health centres had lower attrition than those in lower‐level dispensaries [11, 14]. The decentralization of HIV services to lower‐level dispensaries has increased uptake and patient numbers without considering facility capacity. The higher attrition rates observed in our study could be explained by long waiting times and inadequate services that may lead to reduced defaulter tracing. Task‐shifting using peer mothers can reduce clinic burden and improve ART counselling and retention [41]. ART outreach by community health workers also improves adherence and early defaulter tracing [42]. Additionally, multi‐month ART dispensing can reduce clinic visits and overload, improve quality of care, and hence improve WLHIV retention [43, 44].

Finally, ART attrition was mainly due to the high LTFU of 32% in our cohort, mirroring previous studies from Tanzania and Malawi among women receiving lifelong ART [8, 13, 41]. While the routine data used lack information on the reasons for LTFU, Ugandan and South African studies that traced LTFU women found that a minority (36% vs. 38%) were receiving ART in other facilities [20, 45]. If women classified as LTFU in our study were receiving ART elsewhere, this would lead to an underestimation of the retention rate. The suboptimal registration of deaths among people with HIV complicates disaggregating such cases from LTFU, but given that three‐quarters of women were enrolled without advanced HIV disease, it is likely that a few deaths occurred. The WHO recommends countries to strengthen health information systems that synchronize data across health facilities and service delivery‐points to trace defaulters and lower LTFU rates. Data synchronization would improve long‐term health monitoring for WLHIV on lifelong ART [17].

### 4.1 Strengths and limitations

Strengths of this study include the long follow‐up period (up to 6 years) of a large cohort using data from routine healthcare, allowing attrition rate measurements at different steps throughout the vertical transmission prevention services and chronic HIV care cascades. We used routinely collected programme data from over 90% of Dar es Salaam's health facilities that provide vertical transmission prevention services, making the findings generalizable to similar settings. The data incompleteness of some variables constitutes a limitation but is an unavoidable consequence of using routine healthcare data; however, multiple imputations for missing data yielded similar estimates in the sensitivity analyses. Additionally, some factors previously demonstrated to affect ART retention, such as HIV status disclosure, gravidity, stigma, socio‐economic status, education, mental health and substance abuse, were lacking in the routine data [21, 28]. Our survivor analysis considered a single failure per subject, in which women were censored at ART attrition and hence did not account for women who re‐engaged back into care after LTFU/stopping ART, which may have also underestimated the retention rate. Furthermore, while this study focused on 226 facilities in Dar es Salaam, it is possible that some of the women silently self‐transferred outside the region/study sites and were reported as LTFU, for which we were unable to account. Most participants in this study initiated ART during the index pregnancy. This may not be the case in future studies as the proportion of women who know their HIV status and are on ART increases. Finally, since the study follow‐up period overlapped with the COVID‐19 outbreak, we were unable to determine the effect of the pandemic on the losses observed in our study. Despite these limitations, the current analyses provide valuable evidence to improve our understanding of what needs to be done to improve WLHIV's retention in lifelong ART care following initiation and completion of vertical HIV transmission prevention services.

## 5 CONCLUSIONS

Just over half of WLHIV enrolled in prevention of vertical transmission services and universal lifelong ART were retained on treatment after 6 years of follow‐up. ART attrition was especially high in the first year of enrolment, among adolescent mothers, women enrolled late and those who started ART during pregnancy. Our findings call for targeted interventions to improve ART continuity among WLHIV during and beyond vertical transmission prevention services to end paediatric HIV and keep women and children healthy. These interventions may include strengthening community‐based peer support groups, group ANC counselling, active defaulter tracing via mobile phones and social media, and the use of community healthcare workers [46, 47]. Vertical transmission prevention services and chronic HIV care must optimize the engagement of young women in lifelong ART care and improve community interventions for early ANC attendance. Finally, strengthening national data systems is crucial for health facility data synchronization for defaulter tracing, self‐transfers and long‐term ART outcome monitoring.

## COMPETING INTERESTS

The authors declare no competing interests.

## AUTHORS’ CONTRIBUTIONS

RFU designed the study and wrote the original manuscript under the senior guidance of AEK. RFU and EP performed data abstraction. RFU, GWL and ZZ performed data analysis and interpreted the data with support from AEK, AME, CK and GB. RFU wrote the first manuscript draft, and AEK, AME, GB, CK and GWL provided extensive edits to all manuscript drafts. ZZ, DS, MJM, EP and HN contributed to data interpretation and review of all manuscript versions. PL, AK, KM, BS, AMK and MM contributed to data extraction, data quality assessment and data cleaning, as well as providing critical scientific input on the manuscript. All authors approved the final version of the manuscript for publication.

## FUNDING

RFU received PhD funding from the Swedish International Development Cooperation Agency (SIDA).

## DISCLAIMER

The funder had no role in study design, data collection and analysis, data interpretation, preparation of the manuscript and decision to submit the manuscript for publication.

## Data Availability

Data for this study are from the Tanzanian Ministry of Health, Community Development, Gender Elderly and Children (MOHCDGEC) and are ethically restricted. The authors do not have the ethical or legal right to make an anonymized data set publicity available. The data are available upon request from MOHCDGEC. The point of contact for data request is Prof. Abel Makubi, Permanent Secretary, MOHCDGEC, email ps@afya.go.tz
